# Neighborhood homicide rate and odds of colorectal adenoma among adult patients seeking colonoscopy

**DOI:** 10.1093/jncics/pkae110

**Published:** 2024-10-29

**Authors:** Alyshia Hamm, Evgenia Karayeva, Manoela Lima Oliveira, Nabil Kahouadji, Paul Grippo, Patricia G Wolf, Ece Mutlu, Lisa Tussing-Humphreys, Sage J Kim

**Affiliations:** Department of Kinesiology and Nutrition, College of Applied Health Sciences, University of Illinois Chicago, Chicago, IL 60612, United States; Division of Epidemiology and Biostatistics, School of Public Health, University of Illinois Chicago, Chicago, IL 60612, United States; Department of Kinesiology and Nutrition, College of Applied Health Sciences, University of Illinois Chicago, Chicago, IL 60612, United States; Institute for Health and Research Policy, University of Illinois Chicago, Chicago, IL 60608, United States; Department of Mathematics, Northeastern Illinois University, Chicago, IL 60625, United States; Division of Gastroenterology and Hepatology, Department of Medicine, University of Illinois Chicago, Chicago, IL 60612, United States; University of Illinois Cancer Center, University of Illinois Chicago, Chicago, IL 60612, United States; Department of Nutrition Science, Purdue University, West Lafayette, IN 47907, United States; Division of Gastroenterology and Hepatology, Department of Medicine, University of Illinois Chicago, Chicago, IL 60612, United States; University of Illinois Cancer Center, University of Illinois Chicago, Chicago, IL 60612, United States; Department of Kinesiology and Nutrition, College of Applied Health Sciences, University of Illinois Chicago, Chicago, IL 60612, United States; University of Illinois Cancer Center, University of Illinois Chicago, Chicago, IL 60612, United States; University of Illinois Cancer Center, University of Illinois Chicago, Chicago, IL 60612, United States; Division of Health Policy and Administration, School of Public Health, University of Illinois Chicago, Chicago, IL 60612, United States

## Abstract

**Background:**

Chronic exposure to ambient stressors, including neighborhood crime, may have a detrimental impact on the body’s stress response system with implications for colorectal carcinogenesis.

**Methods:**

We examined associations between the mean neighborhood homicide rates from 2000 and 2018 and diagnosis of colorectal adenoma among patients at the University of Illinois Health and Hospital System in Chicago, Illinois, between 2015 and 2018.

**Results:**

Of the 5225 patients who underwent colonoscopy and were included in the analytic dataset, 60% had colorectal adenoma. Older age, male sex, and higher body mass index (BMI) were associated with greater odds of colorectal adenoma. The neighborhood homicide rate was associated with identifying as Black and Hispanic and higher BMI. A mediation analysis showed that the neighborhood homicide rate effects on colorectal adenoma were mediated through BMI.

**Conclusions:**

The study concluded that older age, male sex, and higher BMI increases the odds of colorectal adenoma, with neighborhood homicide rate indirectly influencing this risk through its association with BMI, particularly among Black and Hispanic individuals.

## Introduction

The American Cancer Society estimates that 106 590 new colorectal cancer (CRC) cases will be diagnosed in the United States in 2024, and 53 010 people will die of the disease.^1^ Approximately 70%-90% of CRC arises from adenomatous polyps, which are neoplastic lesions that harbor a malignant potential and represent an early stage in the development of CRC.^2^ On average, colorectal adenoma is found in 20%-40% of screening colonoscopies.^2^ Among those without germline familial risk, most colorectal adenomas are thought to form through somatic, genetic, and epigenetic modifications stemming from socioenvironmental exposures, such as pollutants, obesity, excess alcohol consumption, combustible tobacco use, poor diet quality, and possibly neighborhood stressors including violent crime.^3-6^

Identifying colorectal adenoma at an early stage and removing the lesion during colonoscopy interrupts the development of CRC.^7^ Efforts to increase uptake and compliance with screening and surveillance in the United States have contributed to a decline in CRC incidence and mortality.^8^ However, this decline has not benefited all demographic groups equally. Colorectal cancer incidence and mortality rates are higher among Black Americans.^9^ Studies have shown that unequal access to CRC screening and quality preventive care, lack of access to and availability of healthy foods, and higher rates of obesity contribute to the CRC health disparity among Black Americans.^10-13^

There are efforts to understand how socioenvironmental exposures promote cancer health disparities. In one analysis, Black residents living in less segregated neighborhoods of Philadelphia, Pennsylvania, were 10% more likely to obtain CRC screening than those living in more segregated neighborhoods.^14^ Furthermore, individuals living in neighborhoods that were characterized as poor or declining were more likely to develop CRC than those living in high-quality or improving neighborhoods.^5^ Notably, neighborhood disadvantages including poverty, segregation, and crime and violence represent chronic stressors that can elicit physiologic responses that augment adipogenic, immune, metabolic, and microbial pathways that are associated with the development of CRC.^5^^,^^14^^,^^15^

**Figure 1.**
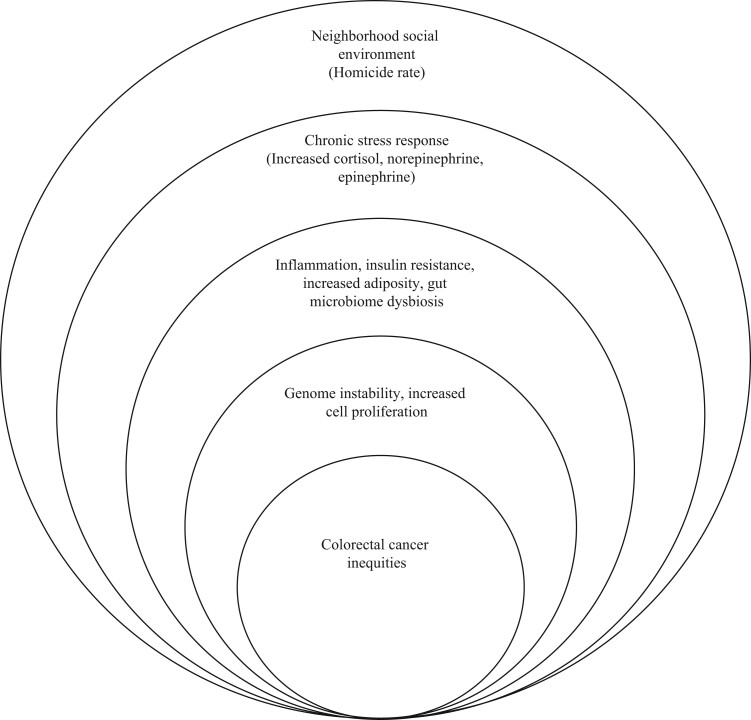
Pathways by which neighborhood social disadvantage can lead to colorectal cancer health inequities.

In response to an acute stressor, the hypothalamic-pituitary-adrenal axis and autonomic nervous system act together.^16^ The autonomic nervous system quickly releases catecholamines, epinephrine, and norepinephrine into circulation. The hypothalamic-pituitary-adrenal axis, however, provides a delayed response, and its final product is the release of the glucocorticoid cortisol. With the release of catecholamines, there is an increase in blood pressure and heart rate,^16^ whereas cortisol promotes an increase of circulating glucose, to support the brain’s increased immune response and subsequent glucose uptake. However, chronic exposure to stress shifts this paradigm (see Figure 1), leading to a consistent increase in epinephrine, norepinephrine, and cortisol can promote increases in overall and central adiposity, increased inflammation, increased insulin resistance, and changes to the composition and metabolic activity of the gut microbiome.^17-19^ Specific to carcinogenesis, chronic stress and downstream immune, metabolic, and microbial dysfunction can promote genome instability and cell proliferation that would support tumor formation.^20^ Moreover, in a recent systematic review of 32 studies, deregulated stress hormones were linked to aberrant DNA methylation patterns that are associated with carcinogenesis.^6^

Neighborhood violent crime is known to affect one’s health.^21^^,^^22^ Furthermore, studies document that stress-related immune responses contribute to many chronic health conditions, including cancer.^23^^,^^24^ Violent crime includes rape, robbery, aggravated assault, and homicide.^25^^,^^26^ Homicide has been used to represent neighborhood violence in previous studies^27^ because homicide events are more likely to be accurately documented in the public domain^28^^,^^29^ compared with other crimes.

The goal of this study was to examine the relationship between neighborhood violence, measured as the homicide rate for a patient’s residential address, and the presence of colorectal adenoma at the time of colonoscopy, the precursor lesion to CRC, using electronic medical record data from the University of Illinois Health and Hospital System (UI Health) in Chicago, Illinois. We hypothesized that exposure to neighborhood violence (high homicide rate) would be associated with a higher incidence of colorectal adenoma and that the strength of the relationship would be strongest among Black patients given the demographic characteristics of neighborhoods most affected by homicide in Chicago. Additionally, we hypothesized that exposure to neighborhood violence would be linked to a higher body mass index (BMI), which is a risk factor for CRC, given a previous report linking community crime in Chicago to excess body weight.^30^ This association is thought to be a result of chronic exposure to stress and dysfunction of the body’s stress system.^19^^,^^31^^,^^32^

## Methods

We retrieved electronic medical record data of patients who underwent a colonoscopy procedure at UI Health between January 1, 2015, and December 31, 2018. UI Health provides health care for underserved populations: approximately 45% of outpatients and 70% of inpatients use some form of public health insurance (eg, Medicaid). UI Health is the only state academic health system that accepts all Medicaid plans and undocumented patients in Illinois. Overall, more than 64% of the patient population is Black or Hispanic individuals, and 46% have a household income of less than $40 000.^33^ The research protocol was reviewed and approved by the University of Illinois Chicago institutional review board (No. 2019-0419). The University of Illinois Chicago Center for Clinical and Translational Science Clinical Bioinformatics Data Warehouse (UL1TR002003) prepared the electronic medical record dataset for the study.

**Figure 2.**
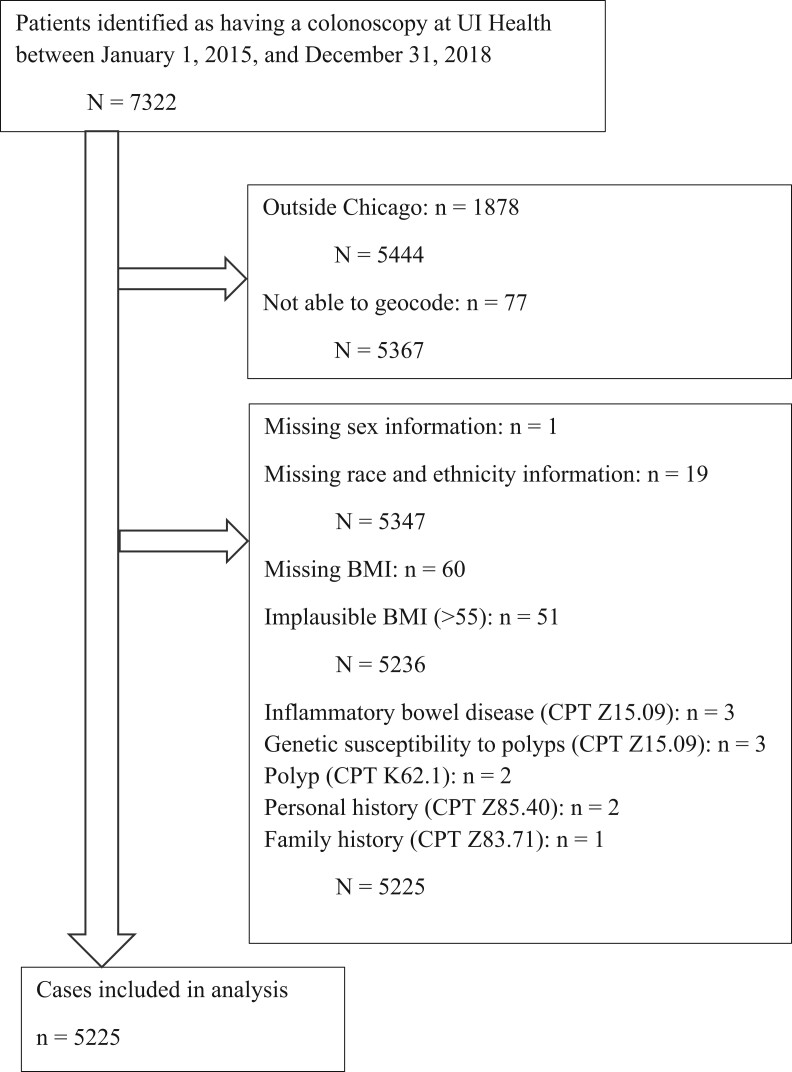
Strengthening the Reporting Observational Studies in Epidemiology diagram. BMI = body mass index; UI Health = Illinois Health and Hospital System.

7322 patient records were retrieved from the UI Health electronic medical record system, who received a colonoscopy based on Current Procedural Terminology Codes (CPT) (see Table S1 for the list of CPT codes used). Figure 2 summarizes the steps of exclusion of patients. Of the 7322 cases with colonoscopy, 5444 patients residing within the city of Chicago were included in this analysis. Of the 5444 patients within Chicago, 77 patients were further excluded because we were not able to locate their residential address within the boundary of Chicago. Of the 5367 geocoded patients, we excluded 1 patient because of missing sex information, and an additional 19 patients were excluded because of missing race and ethnicity information, resulting in a total of 5347 patients. We additionally excluded 60 patients because of missing BMI and an additional 51 cases because of implausible BMI (>55 kg/m^2^). Of these 5236 cases, we further excluded 11 patients because their diagnoses including inflammatory bowel disease, genetic susceptibility to polyps (eg, familial adenomatous polyposis), and inflammatory polyps. This resulted in 5225 unique patients in the analytic dataset. Individual-level variables from the electronic medical record included BMI, age (45 years and older, given CRC screening recommendations changed to 45 years of age in 2017 for Black adults), sex (male or female), and race and ethnicity (Black, Hispanic, and Other races defined as White, Asian, Native American, and Pacific Islander).

The patients’ residential addresses were geocoded, and census tract–level measures were appended. For exposure to violence, we obtained the homicide rates per 100 000 for community areas from the Chicago Health Atlas for years between 2000 and 2018, and average exposure across the years was calculated.^34^ The homicide rate per 100 000 residents was then grouped into high (above the 50th percentile) and low (below the 50th percentile) for the analysis, where the median homicide rate was 26.6 per 100 000 residents. We also added community area–level economic measures (percent poverty and median household income) and access to social services (the rates of food insecurity and having a primary care provider). The rate of food insecurity was measured by percent of residents experiencing limited or uncertain access to adequate food. The rate of having primary care providers was measured by percent residents who report they have at least 1 person they think of as their personal doctor or health-care provider.

Stata 17 (StataCorp, LLC, College Station, TX, USA) was used for statistical analysis. Descriptive statistics were used to summarize the distribution of age, BMI, community-level homicide rate, race and ethnicity, and sex by colorectal adenoma status. χ^2^ tests for categorical comparisons and *t* tests for mean comparisons were used. Multivariate logistic regression was used to explain the likelihood of being diagnosed with colorectal adenoma, adjusting for race and ethnicity, sex, age, and BMI. Statistical significance was determined at a *P* value less than .05 for all analyses. Finally, because the community-level homicide rate is shown to be associated with BMI, and BMI is a known risk factor for colorectal adenoma, we performed a mediation analysis to examine how the association between the community homicide rate and colorectal adenoma may be mediated through BMI.

## Results

Table 1 describes the sociodemographic and clinical characteristics by colorectal adenoma status. 5225 patients residing in 723 census tracts, representing 90.3% of Chicago’s 801 census tracts, were included in the analysis. Of the 5225 patients included in the analysis, 60.1% had a colorectal adenoma. Overall, 55.9% of the patients were Black, 22.4% were Hispanic, and 21.7% were Other race groups including White, Asian, Native American, and Pacific Islander. Male patients were more likely to have colorectal adenoma (*P* < .001). The mean age was 62.3 years for the colorectal adenoma patients and 60.5 years for nonadenoma patients (*P* < .001). Adenoma cases (50.8%) compared with nonadenoma (46.3%) were more likely to be obese with a BMI of at least 30 kg/m^2^ (*P* = .008). There was no statistical difference in homicide rates between colorectal adenoma patients (26.1 per 100 000) and nonadenoma patients (25.6 per 100 000).

**Table 1.** Patient characteristics by colorectal adenoma diagnosis

Characteristics	Total	Colorectal adenoma		*P*
		No, No. (%)	Yes, No. (%)	
Total	5225 (100)	2086 (39.9)	3139 (60.1)	
Race and ethnicity				
Black	2920 (55.9)	1154 (55.3)	1766 (56.3)	
Hispanic	1170 (22.4)	487 (23.3)	683 (21.8)	.396
Other^a^	1135 (21.7)	445 (21.3)	690 (22.0)	
Sex				
Female	2992 (57.3)	1299 (62.3)	1693 (53.9)	<.001
Male	2233 (42.7)	787 (37.7)	1446 (46.1)	
Age				
Mean, y	61.6	60.5	62.3	<.001
Younger than 50	303 (5.8)	176 (8.4)	127 (4.0)	<.001
50-59	2228 (42.6)	964 (46.2)	1264 (40.3)	
60-70	1911 (36.6)	674 (32.3)	1237 (35.7)	
Older than 70	783 (15.0)	272 (13.0)	511 (16.3)	
Body mass index, kg/m^2^				
Mean	30.6	30.4	30.8	<.014
Under, <18.5	76 (1.5)	37 (1.8)	39 (1.2)	<.008
Normal, 18.5-24.9	1025 (19.6)	432 (20.7)	593 (18.9)	
Overweight, 25-29.9	1565 (30.3)	652 (31.3)	913 (29.1)	
Obese, ≥30.0	2559 (49.0)	965 (46.3)	1594 (50.8)	
Homicide rate, per 100 000				
Mean	25.9	25.6	26.1	.313
<50th percentile	2603 (49.8)	1051 (50.4)	1552 (49.4)	.505
≥50th percentile	2622 (50.2)	1035 (49.6)	1587 (50.6)	

a Other race group includes White, Asian, Native American, and Pacific Islander.

Table 2 shows the logistic regression explaining the likelihood of being diagnosed with colorectal adenoma. Male patients were 49% more likely than female patients to have a colorectal adenoma at the colonoscopy (*P* < .001). Patients with obesity were 37% more likely to have colorectal adenoma compared with patients with BMI in the normal range (18.5-24.9 kg/m^2^) (*P* < .001). Race and ethnicity and the neighborhood homicide rate were not statistically associated with the diagnosis of colorectal adenoma.

**Table 2.** Multivariate logistic regression results: odds ratios explaining colorectal adenoma diagnosis

Variable	OR (95% CI)	*P*
Race and ethnicity		
Black	—	—
Hispanic	0.90 (0.77 to 1.07)	.263
Other^a^	1.05 (0.89 to 1.25)	.552
Sex		
Female	—	—
Male	1.48 (1.32 to 1.67)	<.001
Age, y		
Younger than 50	0.55 (0.43 to 0.71)	<.001
50-59	—	—
60-70	1.44 (1.27 to 1.64)	<.001
Older than 70	1.53 (1.29 to 1.82)	<.001
Body mass index, kg/m^2^		
Underweight, ≤18.5	0.73 (0.45 to 1.17)	.190
Normal, 18.5-24.9	—	—
Overweight, 25-29.9	1.05 (0.89 to 1.24)	.541
Obese, ≥30.0	1.37 (1.17 to 1.59)	<.001
Homicide rate, per 100 000		
<50th percentile	—	—
≥50th percentile	1.03 (0.90 to 1.19)	.657
−2 log likelihood	6886.44	

a Other race group includes White, Asian, Native American, and Pacific Islander. Dashes represent ORs that crossed 1.0 - not statistically significant.

Abbreviations: CI = confidence interval; OR = odds ratio.

**Figure 3.**
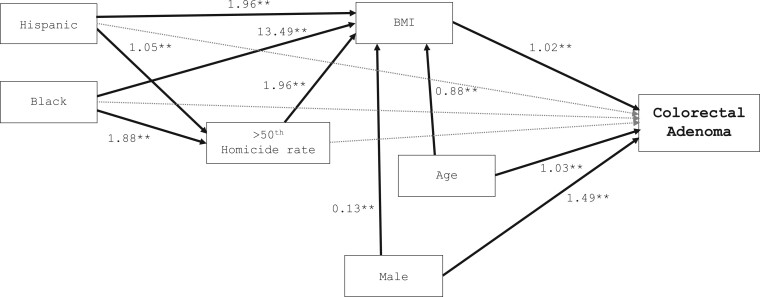
Structural equation model explaining colorectal adenoma diagnosis. Other racial group was used as the referent category. Odds ratios are presented; *P <.05; **P<.01. The **gray dotted line** indicates nonsignificant associations. BMI = body mass index.

We then implemented a structural equation model (Figure 3) to explore the mediation effect of BMI between high homicide and colorectal adenoma. We were particularly interested in exploring racial and ethnic differences in the likelihood of increased BMI among those living in communities with high homicide rates and their relationship to colorectal adenoma. Similar to the logistic regression, higher BMI, older age, and male sex were associated with an increased likelihood of having colorectal adenoma. Living in community areas with a homicide rate above the 50th percentile was not directly associated with colorectal adenoma diagnosis but was associated with higher BMI. We also observed that Black and Hispanic patients were more likely than Other patients to have a higher BMI and were more likely than Other patients to live in communities with homicide rates above the 50th percentile (Table 3).

**Table 3.** Structural equation model exploring the BMI mediation between homicide rate and colorectal adenoma

Variable	Adenoma		BMI		High homicide^b^	
	OR	*P*	OR	*P*	OR	*P*
Age	1.03 (1.02 to 1.03)	<.001	0.89 (0.86 to 0.90)	<.001	—	—
Male	1.49 (1.33 to 1.67)	<.001	0.13 (0.09 to 0.19)	<.001	—	—
Race and ethnicity						
Hispanic	0.87 (0.73 to 1.03)	.118	1.96 (1.24 to 3.10)	<.001	1.05 (1.02 to 1.09)	.002
Black	0.96 (0.81 to 1.14)	.632	13.49 (7.81 to 23.28)	<.001	1.88 (1.83 to 1.93)	<.001
Other race^a^	—	—	—	—	—	—
High homicide^b^	1.04 (0.90 to 1.19)	.615	1.96 (1.24 to 3.10)	<.004	—	—
BMI	1.02 (1.01 to 1.03)	<.001	—	—	—	—

a Other race group was the reference category. Dashes represent ORs that crossed 1.0 - not statistically significant.

b High homicide indicates greater than 2 log likelihood 50th percentile homicide rate.

Abbreviations: BMI = body mass index; OR = odds ratio.

## Discussion

We explored if living in communities with high rates of homicide was associated with the risk of colorectal adenoma among a sample of patients who completed a full access colonoscopy at a large academic medical center in Chicago, Illinois. Our findings indicate that the neighborhood homicide rate was not associated with colorectal adenoma. However, neighborhood homicide was a predictor of colorectal adenoma but mediated through BMI. We also observed that Black patients were significantly more likely than Hispanic, White, Asian, Native American, and Pacific Islanders to live in neighborhoods with high homicide. Moreover, patients with obesity were 37% more likely to have colorectal adenoma at the time of colonoscopy. Obesity is a well-established risk factor for colorectal adenoma and CRC.^35^^,^^36^

We are not the first study to report that neighborhood crime is associated with obesity in Chicago, Illinois. In a previous study, higher neighborhood violent crime was associated with higher rates of obesity in a sample of 15 000 primary care patients in Chicago, Illinois.^30^ Chronic exposure to a stressor like neighborhood violent crime can promote overexpressed cortisol that endorses a diabetogenic state characterized by insulin resistance, lipolysis, hepatic gluconeogenesis, and enhanced and preferential energy storage as fat within visceral adipose tissue .^37^ Chronic stress can also affect eating behavior by inhibiting physiological response to leptin and by increasing ghrelin secretion.^38^ This paradigm would have a direct impact on increasing an individual’s energy intake, which could promote the development of obesity. This suggests that there may be plausible pathways linking neighborhood violent crime to excess body weight—a risk factor for colorectal adenoma.^39^

We further explored obesity distribution by differing age categories. We found notable differences in obesity prevalence between those younger than 60 years and 60 years and older, indicating that the higher prevalence of obesity in the younger age group may be a contributing factor to the increasing incidence of early onset disease among younger individuals. Furthermore, the relationship between younger age and higher BMI was more prominent for Black and Hispanic patients but not for the Other group. However, an increase in early onset CRC has been observed globally. Thus, caution should be exercised in understanding the relations between race and ethnicity obesity and colon carcinogenesis in younger individuals.

Black Americans are disproportionately more likely than other residents to live in disadvantaged neighborhoods affected by poverty, suboptimal built environments, and crime and violence.^40^ It is possible that beyond its deleterious effects on the body’s stress system, living in neighborhoods with high homicide rates may impact other prominent colorectal adenoma risk factors including diet quality and physical activity. Neighborhood violent crime may limit an individual’s ability to procure a high-quality diet and regularly participate in physical activity. However, beyond neighborhood violence and racial and ethnic disparities in health outcomes,^41^ minority communities have shown lower levels of access to care and health-care use. This may be due in part to inadequate health information dissemination^42^ and medical mistrust.^43^

We found that the race and ethnicity of patients were not associated with the incidence of colorectal adenoma. This was somewhat surprising given it is well documented that Black Americans have a higher incidence and mortality of CRC.^10-13^ In Chicago, Illinois, one of the most segregated urban cities in the United States, Black communities are disproportionately affected by multiple stressors and health consequences because of ongoing systemic inequality generated by social, economic, and political structures.^44-51^ Neighborhood environment shapes health risks and outcomes of individuals living within it. Exposure to violent crime can weaken social order and stability within a neighborhood.^52^^,^^53^ The experience of stress from this lack of stability and fear of crime, particularly in the context of increased social and economic deprivation, may negatively affect health through disruption of the body’s stress response with effects on downstream metabolic, immune, and microbial pathways resulting in an increased risk of colon carcinogenesis.^19^^,^^32^^,^^54^^,^^55^ Continuing to examine colorectal adenoma burden through the lens of structural and neighborhood-level exposures and resources will provide critical information about racial disparities in health outcomes and help better distribute screening efforts and resources to the neighborhoods with the highest cancer health burden.

There are several limitations to highlight in this research. First, the neighborhood homicide rate was used as the sole measure of neighborhood stress. Individual-level experiences of stress may provide additional information about how people process and react to social stressors. Because we used electronic medical records for this analysis, we could not know how the actual exposure to neighborhood violence may translate to individual perception or individual-level stress response. Colorectal adenoma development often takes place over a long period of time, suggesting that crime exposures used in the analysis may not have been the most relevant timing of exposure, although we used a mean community homicide rate accounting for data between 2000 and 2018. Research using electronic medical record data has inherent limitations.^56^ Electronic medical record data capture health at a single time point. Additionally, electronic medical record data are fraught with errors.^57^ These errors may include measurement errors (incorrect units of measure, decimal placement, etc), coding errors (wrong CPT code), medication errors, inaccurate health history, misclassification of race and ethnicity, and incomplete information. We were also unable to determine the reason for undergoing colonoscopy beyond screening, which blurs the line between symptomatic patients and those participating in preventive care. Moreover, we are limited to the residential home address provided at the time of colonoscopy. Given this limitation, it is difficult to gauge the chronicity of neighborhood violent crime exposure. Linking to a database like LexisNexis could provide data on residential history and chronicity of the exposure. Another limitation is that our electronic medical record data only represent those who have come to UI Health for a colonoscopy procedure. Individuals living in the most vulnerable communities are less likely to seek preventive medical care, including colonoscopy.^13^ This limits the generalizability of our findings. Finally, our data represent only individuals who live in Chicago, Illinois, and who underwent a colonoscopy at UI Health. Our findings may not be transferrable to other regions or institutions with different patient populations.

Neighborhood homicide rate was not a predictor of odds of colorectal adenoma among adult patients seeking colonoscopy at an academic medical center in Chicago, Illinois. Future studies should consider investigating the relationship between different types of neighborhood crime and colorectal adenoma, as well as explore whether different types of neighborhood social stressors (eg, poor socioeconomic conditions, low social cohesion, food insecurity, lack of access to safe greenspaces) contribute to increased risk. We also encourage that future research focuses on the individual’s perception and experience of stress, using standardized assessments of chronic stress such as the Perceived Stress Scale and cortisol assays that capture chronic stress response. Continuing to build a better understanding of how structural and environmental factors relate to colorectal adenoma has the potential to inform policy makers, hospital administrators, community health workers, and physicians in the targeting of screening initiatives and risk prevention campaigns.

## Supplementary Material

pkae110_Supplementary_Data

## Data Availability

The datasets generated and/or analyzed during the current study are not publicly available due to privacy and confidentiality concerns because this analysis uses individual-level medical records but are available from the corresponding author on reasonable request.
