# Supplementary material for: Neighborhood homicide rate and odds of colorectal adenoma among adult patients seeking colonoscopy
Source: JNCI Cancer Spectr. 2024 Oct 29;8(6):pkae110. doi: 10.1093/jncics/pkae110 (PMC11643352; doi:10.1093/jncics/pkae110)
Supplement: pkae110_Supplementary_Data [file pkae110_supplementary_data.pdf]

**Supplementary Table 1:** CPT codes used to identify procedures

| Code | Description |
|---|---|
| 45380 | Colonoscopy, flexible; with biopsy, single or multiple |
| 45384 | Colonoscopy, flexible; with removal of tumor(s), polyp(s), or other lesion(s) by hot biopsy forceps |
| 45386 | Colonoscopy, flexible; with transendoscopic balloon dilation |
| 45392 | Colonoscopy, flexible; with transendoscopic ultrasound-guided intramural or transmural fine needle aspiration/biopsy(s), includes endoscopic ultrasound examination limited to the rectum, sigmoid, descending, transverse, or ascending colon and cecum, and adjacent structures |
| 45382 | Colonoscopy, flexible; with control of bleeding, any method |
| 45398 | Colonoscopy, flexible; with band ligation(s) (e.g., hemorrhoids) |
| 45381 | Colonoscopy, flexible; with directed submucosal injection(s), any substance |
| 45385 | Colonoscopy, flexible; with removal of tumor(s), polyp(s), or other lesion(s) by snare technique |
| 45379 | Colonoscopy, flexible; with removal of foreign body(s) |
| 45390 | Colonoscopy, flexible; with endoscopic mucosal resection |
| 45389 | Colonoscopy, flexible; with endoscopic stent placement (includes pre- and post-dilation and guide wire passage, when performed) |
| 45379 | Colonoscopy, flexible; with removal of foreign body(s) |
| 45378 | Colonoscopy, flexible; diagnostic, including collection of specimen(s) by brushing or washing, when performed |
| 45388 | Colonoscopy, flexible; with ablation of tumor(s), polyp(s), or other lesion(s) (includes pre- and post-dilation and guide wire passage, when performed) |
| 45391 | Colonoscopy, flexible; with endoscopic ultrasound examination limited to the rectum, sigmoid, descending, transverse, or ascending colon and cecum, and adjacent structures |
| 45393 | Colonoscopy, flexible; with decompression (for pathologic distention) (e.g., volvulus, megacolon), including placement of decompression tube, when performed |
